# Archaeal DNA Polymerase-B as a DNA Template Guardian: Links between Polymerases and Base/Alternative Excision Repair Enzymes in Handling the Deaminated Bases Uracil and Hypoxanthine

**DOI:** 10.1155/2016/1510938

**Published:** 2016-09-19

**Authors:** Javier Abellón-Ruiz, Sonoko Ishino, Yoshizumi Ishino, Bernard A. Connolly

**Affiliations:** ^1^Institute of Cell and Molecular Biology (ICaMB), University of Newcastle, Newcastle upon Tyne NE2 4HH, UK; ^2^Department of Bioscience and Biotechnology, Kyushu University, Fukuoka 812-8581, Japan

## Abstract

In Archaea repair of uracil and hypoxanthine, which arise by deamination of cytosine and adenine, respectively, is initiated by three enzymes: Uracil-DNA-glycosylase (UDG, which recognises uracil); Endonuclease V (EndoV, which recognises hypoxanthine); and Endonuclease Q (EndoQ), (which recognises both uracil and hypoxanthine). Two archaeal DNA polymerases, Pol-B and Pol-D, are inhibited by deaminated bases in template strands, a feature unique to this domain. Thus the three repair enzymes and the two polymerases show overlapping specificity for uracil and hypoxanthine. Here it is demonstrated that binding of Pol-D to primer-templates containing deaminated bases inhibits the activity of UDG, EndoV, and EndoQ. Similarly Pol-B almost completely turns off EndoQ, extending earlier work that demonstrated that Pol-B reduces catalysis by UDG and EndoV. Pol-B was observed to be a more potent inhibitor of the enzymes compared to Pol-D. Although Pol-D is directly inhibited by template strand uracil, the presence of Pol-B further suppresses any residual activity of Pol-D, to near-zero levels. The results are compatible with Pol-D acting as the replicative polymerase and Pol-B functioning primarily as a guardian preventing deaminated base-induced DNA mutations.

## 1. Introduction

Cytosine and adenine bases in DNA can be deaminated to uracil and hypoxanthine, generating U:G and H:T mismatches, which, following replication, lead to mutations in the progeny [1, 2]. Base deamination, a simple hydrolytic reaction accelerated by high temperatures [3], is expected to be especially pronounced in hyperthermophilic organisms, such as many Archaea. As expected, the Archaea possess a number of DNA repair systems dedicated to deaminated bases [4, 5]. Key players include uracil and hypoxanthine DNA glycosylases, which cut the N-glycosidic bond linking these damaged nucleosides to the deoxyribose sugar, initiating base excision repair (BER) [4, 6, 7]. Also present in most Archaea is Endonuclease V (EndoV), which cuts the second phosphodiester bond on the 3′-side of hypoxanthine, beginning alternative excision repair (AER) [8–10]. Recently a novel endonuclease, EndoQ, has been discovered in a subset of Archaea. This enzyme cuts the DNA phosphate 5′ of uracil, hypoxanthine, and abasic sites, again commencing a repair pathway. EndoQ shows activity with the deaminated bases in both single- and double-stranded DNA but abasic sites are only efficiently cut when present in duplex DNA [11, 12]. Recently it has been demonstrated that EndoQ interacts with, and is stimulated by, PCNA [13]. In addition to these DNA repair enzymes, archaeal DNA polymerases possess the unique ability to recognise deaminated bases. Archaeal family-B polymerases (Pol-B) bind tightly to uracil and hypoxanthine and stall replication when these bases are encountered, preventing their copying and transmission of mutations to progeny [14–17]. Interaction with deaminated bases appears confined to archaeal polymerases, not occurring with bacterial or eukaryotic enzymes [18]. Using the enzymes derived from* Pyrococcus furiosus*, interplay between two BER/AER enzymes, Uracil-DNA-glycosylase (UDG) and EndoV, and Pol-B has been investigated [19]. When the polymerase was bound to uracil present in DNA template strands, UDG was inhibited; likewise, polymerase bound to hypoxanthine slowed EndoV. In both cases the presence of PCNA was needed for maximal inhibition. It was proposed that encounter of uracil/hypoxanthine by the polymerase during replication inhibited BER/AER, processes that are inappropriate when these deaminated bases are encountered in single-stranded DNA [19]. In addition to the family-B polymerases, present in all Archaea [20, 21], many members of this domain also possess a family-D enzyme (Pol-D) [20–24]. Gene deletion studies have indicated that, in some archaeal species, Pol-B is dispensable, whereas Pol-D is essential, suggesting that the latter may be the main replicative polymerase [25, 26]. Based on biochemical evidence it has been proposed that Pol-D may act soon after initiation by primase and that at a later stage a switch occurs such that Pol-B becomes responsible for leading strand replication, whereas Pol-D continues to process the lagging strand [27, 28]. More recently* in vitro* experiments have hinted that Pol-D may be responsible for the bulk of genome copying, with Pol-B filling small gaps left by Pol-D as Okazaki fragments are approached [29]. The progression of Pol-D along template strands is slowed by the presence of uracil, by a mechanism yet to be fully clarified but clearly different to that of the family-B enzymes [30]. Very recently it has been demonstrated that hypoxanthine also inhibits Pol-D [31]. In this publication any influence of the family-B and family-D DNA polymerases from* Pyrococcus furiosus* on the activities of UDG, EndoV and EndoQ, as well as interaction between the two polymerases themselves, has been evaluated. It is shown that Pol-B strongly inhibits all three BER enzymes, whereas Pol-D interferes more weakly with these activities. Further, Pol-B abolishes the residual activity that Pol-D demonstrates on uracil-containing templates. These results extend previous observations and give a more complete picture of how these archaeal proteins behave in the presence of deaminated bases [19].

## 2. Materials and Methods

### 2.1. Oligodeoxynucleotide and Protein Preparation

Oligodeoxynucleotides were obtained from ATDBio (Southampton, England) and were desalted and HPLC-purified. The purification of all* Pyrococcus furiosus* proteins has been previously described with appropriate plasmids being used to direct overexpression (in* E. coli*) of the following: Pol-B, wild type, and the 3′-5′ exonuclease minus variant D215A (32); Pol-D, wild type (composing the large and small subunits), and a 3′-5′ exonuclease deficient variant with the mutation H441A in the small subunit (30); PCNA (19); UDG (19); EndoV (9); and EndoQ (11). Primer-templates were prepared by mixing the two single-strands (ratio fluorescent oligodeoxynucleotide : nonfluorescent oligodeoxynucleotide = 1 : 1.25) in 50 mM Tris-HCl pH 8, 100 mM KCl, and heating at 90°C prior to slow cooling to room temperature. The assembled primer-templates were stored frozen at −20°C. *T*_*m*_ values of the primer-templates were measured using a real-time PCR apparatus (Corbett RG-6000). 25 *μ*L of the appropriate DNA (200 nM) in 50 mM Tris-HCl pH 8, 100 mM KCl, was added to 25 *μ*L of 1 : 200 dilution of Quant-iT*™* PicoGreen (Invitrogen). The stock solution of PicoGreen, supplied dissolved in dimethylsulphoxide, was diluted using 50 mM Tris-HCl pH 8, 100 mM KCl. The temperature of the resulting 50 *μ*L solution was increased from 30 to 95°C, over 30 minutes and *T*_*m*_ values determined using the decrease in PicoGreen fluorescence as the DNA strands melted.

### 2.2. Inhibition of BER Enzymes by Pol-B and Pol-D

Any inhibitory influence of the presence of DNA polymerase-B and polymerase-D on the activities of EndoQ, EndoV, and UDG was investigated in 100 *μ*L of 50 mM Tris-HCl pH 8, 100 mM KCl, 1 mM DTT, 1 mM MgCl_2_, and 0.01% (v/v) Tween 20 at 50°C. The primer-template concentration was 10 nM and the following sequence was used (Hex = hexachlorofluorescein and X = thymidine (control), uracil, or hypoxanthine):

 
5′-HEX-GGGGATCCTCTAGAGTCGACCTGC-3′
 
3′- - - - -CCCCTAGGAGATCTCAGCTGGACGACCXTTCGTTCGAACAGAGTACCTGGCTAT-5′

**Figure 1:**
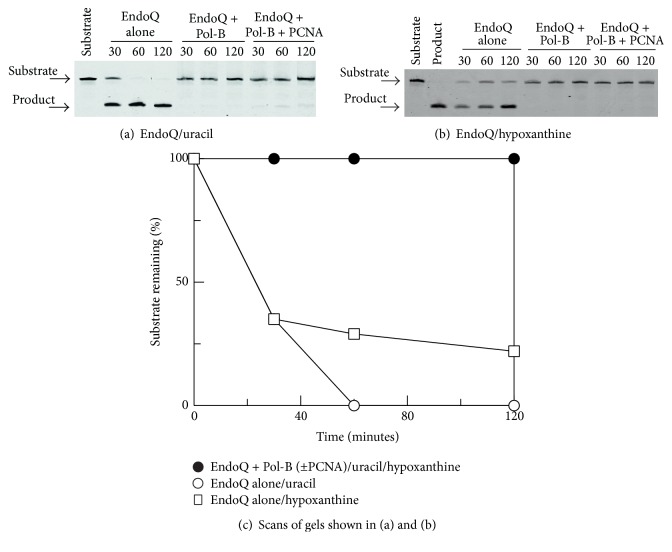
Influence of DNA polymerase-B on the activity of EndoQ. (a) Denaturing gel showing inhibition of EndoQ by Pol-B and Pol-B plus PCNA with uracil. (b) Denaturing gel showing inhibition of EndoQ by Pol-B and Pol-B plus PCNA with hypoxanthine. The numbers above the gel lane indicate the hydrolysis time in minutes. (c) Scans of the gels shown in (a) and (b) indicating the amount of substrate remaining with time. In these experiments Pol-B exo^−^ (D215A) was used. All experiments were repeated at least four times and the inhibition patterns observed were highly reproducible. The data points shown in the scans have an error of <±10%.

**Figure 2:**
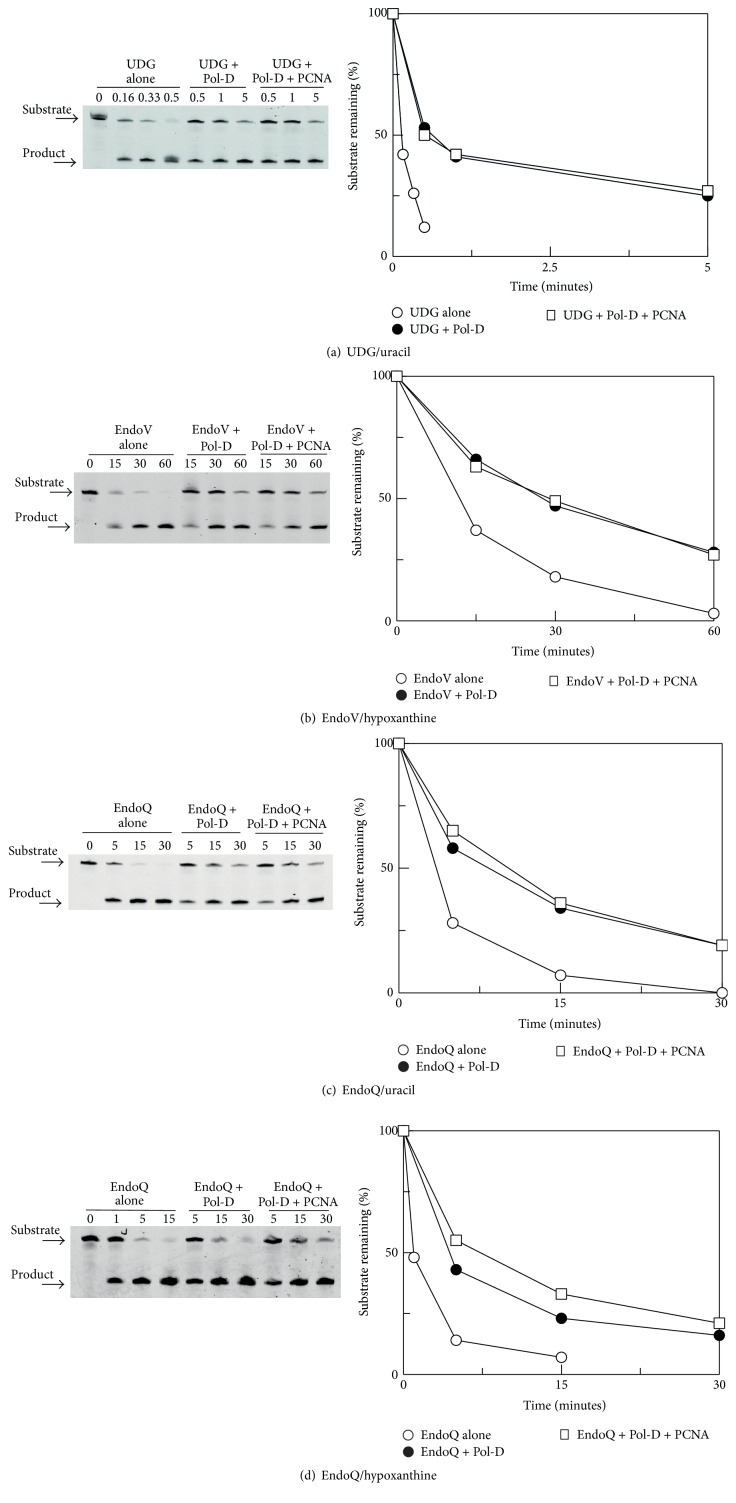
Influence of DNA polymerase-D on the activity of base excision repair enzymes. (a) Inhibition of UDG by Pol-D and Pol-D plus PCNA with uracil. (b) Inhibition of EndoV by Pol-D and Pol-D plus PCNA with hypoxanthine. ((c) and (d)) Inhibition of EndoQ by Pol-D and Pol-D plus PCNA with uracil (c) and hypoxanthine (d). The numbers above the gel lane indicate the hydrolysis time in minutes. In each case a denaturing gel, showing the raw data, and a scan of the gel, showing the substrate remaining with time, are given. In these experiments Pol-D exo^−^ (H445A) was used. These experiments were repeated four times and very similar inhibition was seen in every case. The data points in the scans have an error of <15%.

The levels of Pol-B and PCNA (when used) were 200 nM and reactions were initiated by addition of EndoQ/EndoV or UDG (200 nM). For experiments with EndoQ and EndoV 20 *μ*L aliquots were removed at appropriate times (given in Figures 1 and 2) and the reactions stopped by addition of an equal volume of 95% formamide containing 10 mM EDTA along with a large excess of a “competitor” oligodeoxynucleotide (the competitor sequesters the nonfluorescent component of the duplex, ensuring that the fluorescent DNA runs as a single-strand [16]). The quenched samples were heated at 95°C for 10 minutes and rapidly cooled on ice. 25 *μ*L of the cooled sample was applied to a 17% denaturing (8 M urea) polyacrylamide gel and run at 4 Watts for 2.5 hours. Gels were analysed using a Typhoon FLA9500 imager with ImageQuant software (GE Healthcare). UDG cuts the glycosidic bond of uracil, necessitating an additional treatment under alkaline conditions to develop the strand break. Thus with UDG the reaction was stopped by addition of NaOH (final concentration 0.1 M) followed by heating at 95°C for 15 minutes. Samples were evaporated to dryness using a SpeedVac and redissolved in 20 *μ*L of reaction buffer plus 20 *μ*L of formamide/EDTA/“competitor” prior to analysis as for EndoQ/EndoV.

### 2.3. Inhibition of Pol-D by Pol-B

To investigate any inhibition of DNA polymerase-D by DNA polymerase-B primer-template extensions were performed. Experiments were carried out in 100 *μ*L of 50 mM Tris-HCl pH 8, 100 mM KCl, 1 mM DTT, 1 mM, and 200 *μ*M of each of the 4 dNTPs and 0.01% (v/v) Tween 20 at 50°C. The primer-template concentration was 20 nM and the following sequence, which places uracil 4 bases ahead of the primer-template junction, was used (Cy5 = cyanine-5):

 
5′-Cy5-CCC­ACT­GCA­ATG­GTA­AGT­AAC­GTT­ACG­AGA­TTC­GAG­TCA­TGC­CAG­AAT­TGC­AGGA-3′
 
3′- - - - -GGG­TGA­CGT­TAC­CAT­TCA­TTG­CAA­TGC­TCT­AAG­CTC­AGT­ACG­GTC­TTA­ACG­TCC­TGG­A(T/U)AG­GAA­GAG­ATC­AGA­TCA­ATT­TGG­GTC­AAT­AGG­CTT­ACT­GAC­TGG­ACG­ACCC-5′

**Figure 3:**
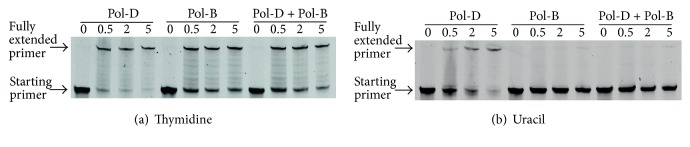
Inhibition of DNA polymerase-D by DNA polymerase-B in the presence of template strand uracil. (a) Extension of a thymidine-containing (control) primer-template by Pol-D, Pol-B, and a mixture of Pol-D and Pol-B. (b) Extension of a uracil-containing primer-template by Pol-D, Pol-B, and a mixture of Pol-D and Pol-B. The numbers above the gel represent the extension time in minutes. In these experiments wild type (exo^+^) Pol-D and Pol-B were used. These experiments were carried out three times and in every case complete inhibition of Pol-D by Pol-B was noticed when uracil was present in the template.

Reactions were initiated by addition of polymerase (200 nM) (when Pol-B and Pol-D were used together the proteins were added simultaneously and run for the times shown in Figure 3). To terminate the reaction a 20 *μ*L aliquot was added to an equal volume of 95% formamide containing 10 mM EDTA and the mixture heated at 95°C for 10 minutes prior to rapid cooling on ice. 25 *μ*L of the cooled sample was applied to a 17% denaturing (8 M urea) polyacrylamide gel and run at 4 Watts for 2.5 hours. This gel was heated to about 50°C by circulating water, the elevated temperature ensuring separation of the two strands. The gels were analysed as given above for the EndoQ/EndoV/UDG experiments.

## 3. Results

### 3.1. Inhibition of EndoQ by Pol-B

Although it is known that Pol-B suppresses UDG and EndoV activity [19], EndoQ had yet to be discovered when these experiments were carried out. Any influence of Pol-B on the ability of EndoQ to cut DNA containing uracil or hypoxanthine use was assessed using a chemically synthesised primer-template (Figure 1). This substrate, comprising a 24-base primer annealed to a 54-base template, locates the damaged base at the +4 position in the template, the site at which Pol-B interacts most strongly with uracil and hypoxanthine [14]. All investigations, with these thermophile-derived enzymes, were carried out at 50°C. However, the primer-template demonstrated *T*_*m*_ of 77.0 ± 0.2°C under the buffer conditions used (data not shown) and, therefore, exists predominantly in the double-stranded form. A Pol-B derivative disabled in proof-reading exonuclease activity, D215A, was used for this experiment [32], which involved long incubations of the primer-templates with relatively high concentrations of polymerase in the absence of dNTPs. When the wild type, exo^+^, variant was used some degradation of the fluorescent template was observed due to proof-reading activity, which obfuscated the results. The exo^−^ D215A variant is not compromised in deaminated base binding [14, 15]. When the uracil-containing primer-template was incubated with EndoQ, complete hydrolysis was observed after 1 hour (Figure 1(a)). In the case of hypoxanthine about 75% of the substrate was destroyed between 1 and 2 hours (Figure 1(b)). Repeating these experiments in the presence of Pol-B showed that both the uracil and hypoxanthine substrates were fully stable for 2 hours (Figures 1(a) and 1(b)). Clearly the presence of Pol-B almost completely abolishes the activity of EndoQ at both uracil and hypoxanthine; so profound is the inhibition that there is little room for additional improvement in the presence of PCNA (Figures 1(a) and 1(b)). Scans of these gels more plainly illuminate the massive degree of shielding offered by Pol-B (Figure 1(c)).

### 3.2. Inhibition of UDG, EndoV, and EndoQ by Pol-D

Replication by archaeal DNA polymerase-D is inhibited when uracil or hypoxanthine is present in template strands, suggesting that this enzyme, like Pol-B, is able to specifically recognise deaminated bases [30, 31]. We wondered, therefore, if Pol-D interferes with DNA repair enzymes that process these two bases, in an analogous fashion to the Pol-B mediated inhibition of EndoQ, UDG, and EndoV [19]. For these investigations the primer-template, employed above for the experiments with Pol-B, placing uracil/hypoxanthine at +4, was used. However, Pol-D is much more tolerant than Pol-B with regard to damaged base location, being inhibited by uracil up to 100 bases ahead of the primer-template junction [30]. The influence that the presence of Pol-D has on the activities of the base excision repair enzymes UDG, EndoV, and EndoQ is shown in Figure 2. Here gels showing the primary data along with scans of the gels, giving the amount of the substrate primer-template remaining with time, are illustrated. Once again a Pol-D variant lacking proof-reading exonuclease activity (H445A) was used [30]. The results are very similar for the four combinations tested; UDG/uracil (Figure 2(a)), EndoV/hypoxanthine (Figure 2(b)), EndoQ/uracil (Figure 2(c)), and EndoQ/hypoxanthine (Figure 2(d)). In each instance inclusion of Pol-D slows the rate at which the repair enzymes act on their substrates by a measurable amount. For all the investigations presented in Figure 2, an additional experiment exploring the impact of PCNA, added along with Pol-D, was carried out. However, any influence of PCNA was very marginal and this protein barely enhances the shielding offered by Pol-D alone. This contrasts with earlier results seen with Pol-B and UDG/EndoV; here PCNA substantially enhances the protection afforded by the polymerase [19]. While Pol-D does inhibit UDG, EndoV, and EndoQ, it is clear that it affords substantially less protection than does Pol-B. This is unmistakable when comparing the influences of Pol-B and Pol-D on EndoQ activity described in this publication; Pol-B almost completely inhibits EndoQ; Pol-D merely slows the enzyme (Figures 1, 2(c), and 2(d)). UDG and EndoV behave similarly; an earlier publication showed profound inhibition by Pol-B [19], contrasting with the more modest influence of Pol-D seen here (Figures 2(a) and 2(b)).

### 3.3. Inhibition of Pol-D by Pol-B

The copying of DNA by both archaeal Pol-B and Pol-D is hampered by the presence of template strand uracil, with the base acting more potently on Pol-B [14–17, 30]. Given the strong suppression of UDG, EndoV, and EndoQ activities by Pol-B, we wondered if Pol-B might further retard the, already hindered, progression of Pol-D through uracil-containing templates. A control primer-template, lacking uracil, was efficiently elongated by both Pol-B and Pol-D, with a mixture of the two enzymes behaving similarly to the individual components (Figure 3(a)). With a primer-template having single uracil four bases ahead of the primer-template junction, Pol-D is able to generate fully extended product (Figure 3(b)). As expected, though, the rate of copying of the uracil-containing DNA is slower than that observed with the control. In contrast Pol-B is totally unable to copy the uracil-bearing DNA, emphasising the more powerful inhibition uracil exerts on this enzyme. With a mixture of Pol-D and Pol-B, no polymerisation at all is observed, clearly illustrating that Pol-B suppresses any residual activity that Pol-D displays towards templates in which uracil is present (Figure 3(b)).

## 4. Discussion

Repair enzymes, acting on uracil and hypoxanthine, and DNA polymerases are common to all organisms but, unusually, archaeal polymerases in the B and D families are also able to sense deaminated bases [14–17, 30, 31]. This overlap in substrate specificity raises questions about enzyme interplay and earlier Pol-B was observed to inhibit UDG (at uracil) and EndoV (at hypoxanthine), with PCNA enhancing the effect [19]. This publication increases our knowledge about how archaeal BER/AER enzymes and polymerases interact. It is shown that Pol-B strongly suppresses the activity of EndoQ at both uracil and hypoxanthine, the inhibition being so profound that PCNA has little chance of further potentiation. We extend observations to Pol-D, demonstrated here to slow UDG, EndoV, and, to a lesser extent, EndoQ. In general Pol-D appears to be a weaker inhibitor of base excision repair enzymes compared to Pol-B and, at least with UDG and EndoV, PCNA plays little additional role. Finally Pol-B is seen to almost completely retard the progression of Pol-D along uracil-containing templates. Pol-D is itself directly inhibited by uracil in DNA but the presence of Pol-B strongly potentiates the influence of the deaminated base.

It is most likely that the profound inhibition of UDG, EndoV, EndoQ, and Pol-D arises from the tight and specific binding of Pol-B to deaminated bases, which strongly shields the DNA from the action of other enzymes. Complexes between these enzymes have not been detected in high throughput screens, suggesting little direct role for protein-protein interactions in the inhibition patterns observed [33, 34]. Previously it has been demonstrated that Pol-B binds to uracil-containing primer-templates with *K*_*D*_ values around 1 nM [14] and that hypoxanthine interacts 1.5–4.5 times more weakly [35]. Control primer-templates bind 1000-fold less strongly [14, 35], suggesting that much of the binding is mediated through strong interactions with the deaminated bases. At the 200 nM levels of Pol-B used throughout these studies, full binding of the DNA substrates is expected. As structures of Pol-B with bound uracil and hypoxanthine, which confirm the profound interactions with these bases, have been determined [15, 17], its inhibitory action can be rationalised. Considering the DNA template strand, Pol-B effectively shields the glycosidic bond of the deaminated base (target for UDG), the neighbouring 5′-phosphate (target for EndoQ), and the phosphodiester two positions in the 3′ direction (target for EndoV). The occlusion of the 5′-phosphate, to a highly conserved tyrosine [15], is especially pronounced, explaining the very profound inhibition of EndoQ. Alternatively, this may simply arise as higher concentrations of Pol-B (200 nM) were used here than in the earlier investigations with UDG and EndoV (100 nM). In the case of the primer, Pol-B occludes the 3′-OH of the final deoxynucleotide, the initiation point for Pol-D.

Pol-D also inhibits UDG, EndoV, and EndoQ but to a noticeably lesser extent than Pol-B. An earlier study showed that Pol-D bound primer-templates containing uracil with *K*_*D*_ around 5 nM, again inferring that Pol-D levels of 200 nM should be sufficient for complete saturation of the primer-templates (30). However, there is a crucial difference between the manners in which Pol-B and Pol-D interact with deaminated bases. With Pol-B controls bind 1000-fold more weakly, compatible with strong interaction with deaminated bases. With Pol-D controls bind only 2 times less strongly (30), suggesting much of the binding is to general features of the DNA with only marginal additional interactions with the deaminated base. This feature both explains why Pol-D catalysed extension is less sensitive to the presence of deaminated bases and is consistent with the lower inhibition of BER/AER enzymes seen with Pol-D versus Pol-B. Unfortunately, no structures are yet available for Pol-D and so the nature of the inhibition of BER enzymes cannot be rationalised to the same extent as Pol-B. Presumably though simple steric hindrance, albeit much less pronounced than observed with Pol-B, underlies Pol-D mediated inhibition of BER proteins.

**Figure 4:**
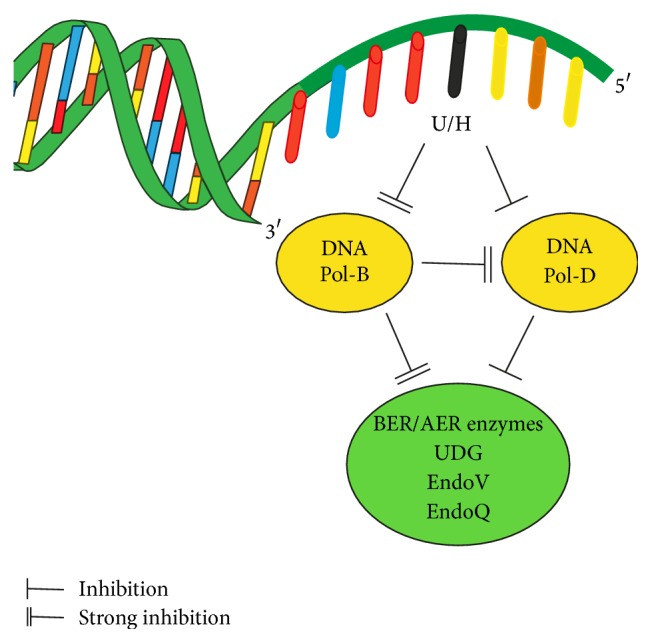
Summary of the interplay between archaeal DNA polymerases, base excision repair enzymes, and template strand deaminated bases.

Overall the interplay between DNA deaminated bases, archaeal DNA polymerases, and base excision repair enzymes is summarised in Figure 4. Based on these observations we suggest that Pol-D is present in the replisome and responsible for the majority of* de novo* synthesis of both leading and lagging strands [29]. Pol-B does not function as a replicative enzyme but serves primarily to guard against deaminated base-induced mutations. A similar scenario has recently also been proposed based on the interaction of EndoQ with PCNA [13]. To fulfil such a role Pol-B may be a replisome component, positioned ahead of Pol-D ready to intercept uracil and hypoxanthine. However, Pol-B seems to make few direct interactions with replisome components [33, 34]. More likely Pol-B, a relatively abundant archaeal protein [26], binds these bases directly from solution as they are rendered single-stranded by the action of the replicative helicase. The slowing of Pol-D as it approaches these bases would increase the chances of Pol-B binding prior to Pol-D arrival. Once bound to uracil or hypoxanthine Pol-B profoundly inhibits Pol-D catalysed genome copying, stalling the replication fork and allowing regression to a “chicken-foot” structure [5]. Such a manoeuvre temporarily restores the deaminated bases to their original double-stranded location to allow accurate BER/AER. The exclusion of BER/AER enzymes from deaminated bases in single-stranded templates would buy time for fork regression and also, by preventing repair in single-strands, avoid strand scission and permanent separation of DNA ends. BER/AER in any context, including that described here, requires limited amounts of DNA synthesis for completion of the repair process and it is here that the polymerase activity of Pol-B is likely to come into play. Thus a simple uracil/hypoxanthine binding protein to inhibit replication and repair would be a less efficient solution compared to a polymerase with the additional functionality.
